# Comparative modelling of a novel enzyme: Mus musculus leucine decarboxylase

**DOI:** 10.3906/kim-2003-63

**Published:** 2020-06-01

**Authors:** Arif Sercan ŞAHUTOĞLU

**Affiliations:** 1 Department of Chemistry, Faculty of Science and Arts, Canakkale Onsekiz Mart University, Canakkale Turkey

**Keywords:** Homology modelling, leucine decarboxylase, isopentylamine, Gm853, ornithine decarboxylase

## Abstract

Leucine decarboxylase (LDC) is a recently proposed enzyme with no official enzyme commission number yet. It is encoded by the Mus musculus gene Gm853 which is expressed at kidneys, generating isopentylamine, an alkylmonoamine that has not been described to be formed by any metazoan enzyme yet. Although the relevance of LDC in mammalian physiology has not been fully determined, isopentylamine is a potential modulator which may have effects on insulin secretion and healthy gut microbiota formation. The LDC is a stable enzyme that specifically decarboxylates L-leucine but does not decarboxylate ornithine or lysine as its paralogues ornithine decarboxylase (ODC; EC: 4.1.1.17) and lysine decarboxylase (KDC; EC: 4.1.1.18) do. It does not act as an antizyme inhibitor and does not decarboxylate branched amino acids such as valine and isoleucine as it is another paralogue valine decarboxylase (VDC; EC: 4.1.1.14). The crystal structure of the enzyme has not been determined yet but there are homologous structures with complete coverage in Protein Data Bank (PDB) which makes LDC a good candidate for comparative modelling.In this study, homology models of LDC were generated and used in cofactor and substrate docking to understand the structure/function relationship underlying the unique selectivity of LDC enzyme.

## 1. Introduction

Polyamines are biologically important poly-cations that are essential for many important biological processes including protein and nucleic acid synthesis, native structure formation, protection from oxidative damage, block and modulation of ion channels, cell proliferation, cell differentiation, and apoptosis [1]. Due to their crucial function in the cell, polyamine levels are strictly controlled through various mechanisms. Ornithine decarboxylase (ODC; EC 4.1.1.17) is the first enzyme in the polyamine synthesis [2] and is a member of group IV amino acid decarboxylases which is a subset of pyridoxal-5’-phosphate (PLP)-dependent enzymes that participate in the generation of biogenic amines or neurotransmitters [3]. ODC catalyses the decarboxylation of ornithine (Orn, O), a product of the urea cycle, to form putrescine. This reaction is the committed step in polyamine synthesis.

ODC works as a homodimer in a constant state of association and dissociation. The exchange of homodimer subunits enables regulation by an autoregulatory loop that involves proteins called antizymes (AZs) and antizyme inhibitors (AZINs). AZs are polyamine induced proteins that bind ODC subunits with higher affinity than these subunits themselves. The formation of the ODC:AZ heterodimers results not only in the inactivation of ODC, but also in targeting for ubiquitin independent degradation by proteasomes. AZs themselves are regulated by other proteins called AZINs, that are highly homologous to ODC, but lack decarboxylase activity [2].

Leucine decarboxylase (LDC) is a recently proposed amino acid decarboxylase [3]. LDC is an ornithine decarboxylase paralogue with a specific leucine decarboxylase activity. Unlike similar enzymes, LDC acts on leucine but not ornithine, lysine (Lys, K) valine (Val, V), or isoleucine (Ile, I) and does not act as an antizyme inhibitor. In this aspect, LDC is different from lysine decarboxylase (KDC; EC 4.1.1.18) and valine decarboxylase (VDC; EC 4.1.1.14) which was synonymously known as leucine decarboxylase due to its activity on leucine till now [4]. LDC is one of the 3 known ODC-related proteins together with AZIN1 and AZIN2 and suspected as a third AZIN [2] until recently.

The enzyme catalyses the formation of isopentylamine (isoamylamine), a product with a role and metabolism that is not fully understood in mammals yet. Human breastmilk, meconium and infant’s faces are known to contain isopentylamine which may contribute to facilitating the formation of healthy gut microbiota [5]. Moreover, a group of trace amine-associated receptors (TAAR) family is reported to be activated by trace amines, isopentylamine, 2-phenylethylamine, p-tyramine, and agmatine which significantly increases intracellular cAMP. This activation is reported to result in increased insulin secretion in mouse under normal conditions but not in glucolipotoxicity thus causing dysregulated TAAR signalling in type 2 diabetes[6].

In this study, comparative models of LDC is generated and used to investigate the cofactor and substrate interaction for gaining a better understanding on the structure/function relationship and unique substrate selectivity of the novel enzyme.

## 2. Materials and methods

### 2.1. Database search and sequence alignment

LDC target sequence homologs in different databases were searched using The Basic Local Alignment Search Tool (BLAST) [7]. The multiple sequence alignments were performed with Clustal-Omega software [8]. Easy Sequencing in PostScript (ESPript-3.0) [9] program was used to render sequence similarities and secondary structure information from aligned sequences. Phylogenetic trees were prepared and rendered using Molecular Evolutionary Genetics Analysis (MEGA) software. Sequence logos of the alignments were prepared with WebLogo 3 web-based application [10].

### 2.2. Comparative modelling

The crystal structure of human ODC (PDB: 1D7K) was used as template [11]. The homology model of the enzyme was generated using the SWISS-MODEL protein structure homology-modelling server [12]. The reliabilities of the template and the model were verified by The Structure Analysis and Verification Server (SAVEShttp:// servicesn.mbi.ucla.edu/SAVES/) of UCLA-DOE-LAB using PROCHECK [13], WHATCHECK [14], ERRAT [15], VERIFY 3D [16,17], and PROVE [18] programs. Structural Alignment of Multiple Proteins (STAMP) program of MultiSeq module of Visual Molecular Dynamics (VMD; http://www.ks.uiuc.edu/Research/vmd/) was used to superpose the structures [19]. Structures of templates and models were analysed and summarized with Pictorial Database of 3D structures in the Protein Data Bank (PDBsum) [20].

### 2.3. Docking of lig

Docking of ligands was performed with SwissDock (http://www.swissdock.ch/) [21] molecular docking server based on docking software EADock DSS [22]. The pyridoxal-5-phosphate (PLP) and leucine (Leu, L) ligand structures were derived from ZINC database (http://zinc.docking.org/) [23]. Drawing, displaying, and characterizing of ligands were performed with Marvin 17.6, 2017, ChemAxon (http://www.chemaxon.com). Docking preparation for protein model and evaluation of dock results were performed with UCSF-Chimera (http://www.rbvi.ucsf.edu/chimera) software developed by the Resource for Biocomputing, Visualization, and Informatics at the University of California, San Francisco [24]. Molecular Dynamics (MD) simulations were run for 10 nanoseconds (ns) for minimization and equilibration purposes of the models and the complexes. The computational resources are provided by TÜBİTAK ULAKBİM, High Performance and Grid Computing Centre (TR-Grid e-Infrastructure). 2D representations of docked ligands were prepared with PoseView [25] available via Hamburg University Centre for Bioinformatics (ZBH) website (https://proteins.plus/).

## 3. Results

### 3.1. LDC is a type III PLP-dependent enzyme

**Figure 1:**
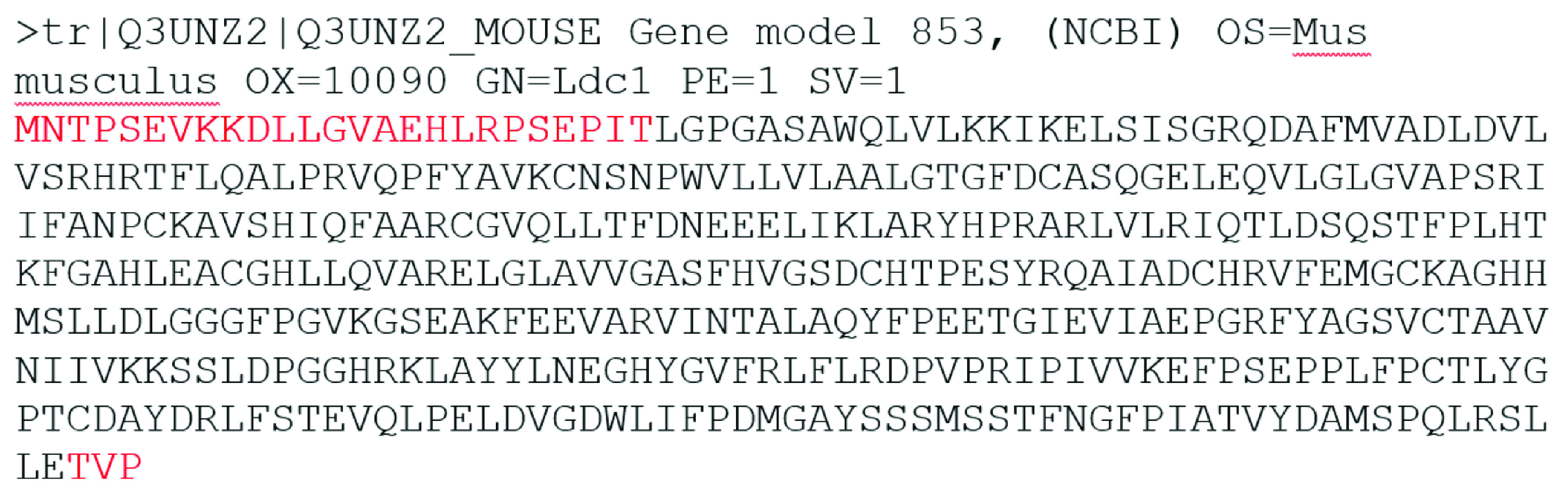
The LDC sequence in fasta format. Red residues have not included in full models due to the lack of coverage in the template file.

**Figure 2:**
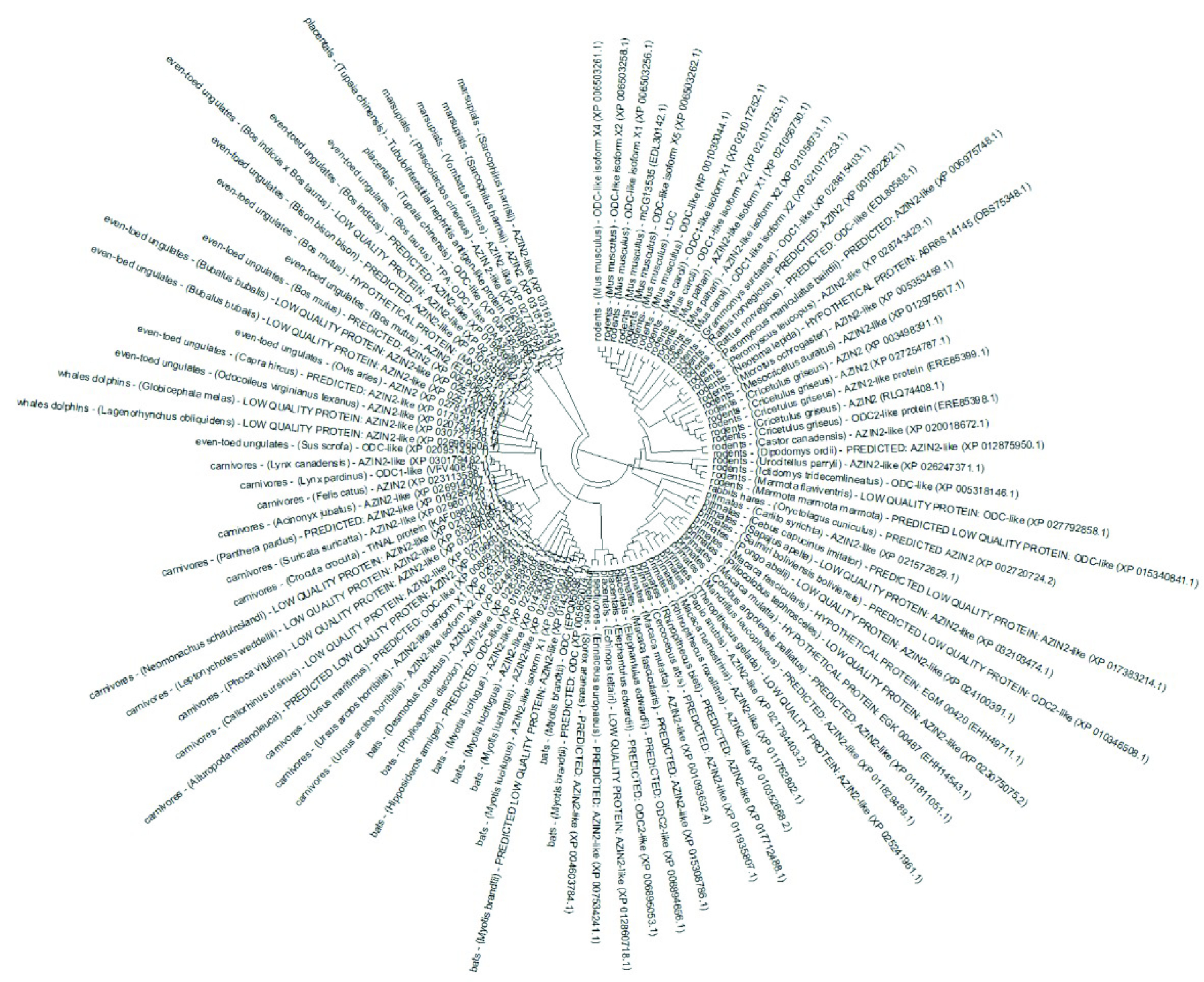
Phylogenetic tree for LDC sequence prepared with fast minimum evolution method.

The initial database searches for LDC sequence (Figure 1) is performed by using BLASTp program and BLOSUM62 matrix in nr and pdb data set. The sequences producing significant alignments are given in Table 1 and Table 2, respectively. Phylogenetically, LDC sequence is most similar to rodent, primate, rabbit & hare, bat, whale & dolphin, placental, insectivore, carnivore, marsupial, and even-toed ungulate ornithine decarboxylase 1-like and antizyme inhibitor 2-like proteins (Figure 2). Most of the sequences in Table 1 are not investigated in protein level meaning that there may be several LDC isoenzymes that wait for characterization in these organisms.

**Table 1:** Alignment results for LCD sequence in nonredundant protein sequences database.

Description	Total score	Query cover	E value	Percent identity	Accession
ODC-like [ *Mus musculus* ]	875	100%	0.0	100%	NP_ 001030044.1
ODC1-like isoform X1 [ *Mus caroli* ]	840	100%	0.0	95.56%	XP_ 021017252.1
AZIN2-like isoform X1 [ *Mus pahari* ]	836	100%	0.0	95.29%	XP_ 021056730.1
ODC-like isoform X1 [ *Mus musculus* ]	786	90%	0.0	99.48%	XP_ 006503256.1
ODC-like isoform X2 [ *Mus musculus* ]	785	90%	0.0	99.48%	XP_ 006503258.1
ODC-like isoform X4 [ *Mus musculus* ]	785	90%	0.0	99.48%	XP_ 006503261.1
AZIN2-like [ *Mastomys coucha* ]	765	100%	0.0	88.00%	XP_ 031232770.1
Predicted: AZIN2 [ *Rattus norvegicus* ]	758	100%	0.0	86.35%	XP_ 001062262.1
ODC1-like [ *Grammomys surdaster* ]	757	100%	0.0	86.12%	XP_ 028615403.1
AZIN2-like isoform X2 [ *Mus pahari* ]	746	90%	0.0	94.27%	XP_ 021056731.1
Predicted: AZIN2-like [ *Rattus norvegicus* ]	727	98%	0.0	84.65%	EDL80588.1
Predicted: AZIN2-like [ *Peromyscus maniculatus bairdii* ]	719	98%	0.0	82.97%	XP_ 006975748.1
AZIN2-like [ *Peromyscus leucopus* ]	717	100%	0.0	81.88%	XP_ 028743429.1
AZIN2-like [ *Microtus ochrogaster* ]	704	100%	0.0	80.24%	XP_ 005353459.1
ODC1-like isoform X2 [ *Mus caroli* ]	699	83%	0.0	96.05%	XP_ 021017253.1
AZIN2 [ *Cricetulus griseus* ]	698	100%	0.0	79.76%	XP_ 027254787.1
AZIN2 [ *Cricetulus griseus* ]	694	100%	0.0	79.53%	XP_ 003498391.1
Hypothetical protein: A6R68_ 14145 [ *Neotoma lepida* ]	692	98%	0.0	81.10%	OBS75348.1
mCG13535 [ *Mus musculus* ]	692	83%	0.0	96.06%	EDL30142.1
AZIN2 [ *Cricetulus griseus* ]	687	98%	0.0	79.67%	RLQ74408.1
ODC2-like protein [ *Cricetulus griseus* ]	684	100%	0.0	78.82%	ERE85398.1
AZIN2-like [ *Mesocricetus* *auratus* ]	682	100%	0.0	78.35%	XP_ 012975617.1
AZIN2-like [ *Castor canadensis* ]	652	100%	0.0	75.06%	XP_ 020018672.1
AZIN2-like [ *Carlito syrichta* ]	634	100%	0.0	73.41%	XP_ 021572629.1
ODC-like [ *Ictidomys tridecemlineatus* ]	622	99%	0.0	73.77%	XP_ 005318146.1
ODC 2-like protein [ *Cricetulus griseus* ]	622	90%	0.0	79.17%	ERE85399.1
Low quality protein: ODC-like [ *Marmota flaviventris* ]	619	99%	0.0	73.72%	XP_ 027792858.1
Predicted: AZIN2-like [ *Macaca fascicularis* ]	615	95%	0.0	74.38%	XP_ 015308786.1
Predicted: AZIN2-like [ *Dipodomys ordii* ]	613	95%	0.0	75.80%	XP_ 012875950.1
AZIN2-like [ *Urocitellus parryii* ]	613	95%	0.0	75.24%	XP_ 026247371.1
AZIN2-like isoform X2 [ *Ursus arctos horribilis* ]	612	100%	0.0	70.59%	XP_ 026372613.1
Predicted: AZIN2 [ *Oryctolagus cuniculus* ]	612	99%	0.0	71.39%	XP_ 002720724.2
Predicted: AZIN2-like [ *Cercocebus atys* ]	612	95%	0.0	73.89%	XP_ 011935807.1
AZIN2-like [ *Macaca mulatta* ]	611	95%	0.0	73.89%	XP_ 001093632.4
AZIN2-like [ *Myotis lucifugus* ]	610	94%	0.0	75.12%	XP_ 014305039.2
Predicted: AZIN2-like [ *Mandrillus leucophaeus* ]	608	94%	0.0	74.31%	XP_ 011829489.1
Hypothetical protein: EGM_ 00420 [ *Macaca fascicularis* ]	607	94%	0.0	74.06%	EHH49711.1
AZIN2-like [ *Papio anubis* ]	606	94%	0.0	74.06%	XP_ 021794403.2
AZIN2-like [ *Macaca nemestrina* ]	606	94%	0.0	74.06%	XP_ 011762802.1
Hypothetical protein: EGK_ 00487 [ *Macaca mulatta* ]	605	94%	0.0	74.06%	EHH14543.1
AZIN2 [ *Felis catus* ]	605	97%	0.0	71.15%	XP_ 023113588.1
AZIN2-like isoform X1 [ *Ursus arctos horribilis* ]	604	100%	0.0	68.81%	XP_ 026372610.1
Predicted: ODC-like [ *Ursus maritimus* ]	603	98%	0.0	70.57%	XP_ 008693048.1
AZIN2-like [ *Suricata suricatta* ]	602	98%	0.0	70.50%	XP_ 029801720.1
Low quality protein: AZIN2-like [ *Theropithecus* *gelada* ]	601	94%	0.0	73.57%	XP_ 025241961.1
AZIN2-like [ *Myotis* *lucifugus* ]	601	93%	0.0	74.75%	XP_ 023599999.1
AZIN2-like *[Rhinopithecus roxellana* ]	598	95%	0.0	72.41%	XP_ 010352668.2
AZIN2-like [ *Myotis lucifugus* ]	597	93%	0.0	73.80%	XP_ 023600018.1
Predicted: AZIN2-like [ *Rhinopithecus bieti* ]	596	94%	0.0	72.57%	XP_ 017712488.1
Predicted: ODC-like [ *Hipposideros armiger* ]	593	95%	0.0	71.43%	XP_ 019513209.1
AZIN2-like [ *Acinonyx jubatus* ]	591	98%	0.0	71.60%	XP_ 026914007.1
Low quality protein: AZIN2-like [ *Piliocolobus tephrosceles* ]	591	94%	0.0	72.07%	XP_ 023075075.2
Predicted: ODC [ *Myotis brandtii* ]	588	93%	0.0	73.30%	XP_ 005862074.2
Predicted low quality protein: ODC-like [ *Marmota* *marmota marmota* ]	587	98%	0.0	70.90%	XP_ 015340841.1
ODC1-like [ *Lynx pardinus* ]	585	99%	0.0	70.05%	VFV40845.1
AZIN2-like isoform X1 [ *Myotis lucifugus* ]	585	94%	0.0	72.14%	XP_ 023600014.1
ODC [ *Myotis brandtii* ]	585	93%	0.0	72.10%	EPQ05098.1
Predicted: AZIN2-like [ *Panthera pardus* ]	584	97%	0.0	71.63%	XP_ 019285295.1
ODC-like [ *Sus scrofa* ]	584	99%	0.0	67.14%	XP_ 020951430.1
Predicted: AZIN2-like [ *Capra hircus* ]	583	95%	0.0	69.95%	XP_ 017921811.1
Predicted: AZIN2-like [ *Colobus angolensis palliatus* ]	582	94%	0.0	71.39%	XP_ 011811051.1
Predicted: AZIN2-like [ *Erinaceus europaeus* ]	582	93%	0.0	71.03%	XP_ 007534241.1
AZIN2-like [ *Lynx canadensis* ]	579	97%	0.0	70.43%	XP_ 030179482.1
Low quality protein: AZIN2-like [ *Pongo abelii* ]	575	94%	0.0	72.57%	XP_ 024100391.1
AZIN2 [ *Ovis aries* ]	572	94%	0.0	68.63%	XP_ 027820674.1
Predicted: ODC2-like [ *Elephantulus edwardii* ]	572	96%	0.0	66.83%	XP_ 006894656.1
AZIN2-like [ *Phyllostomus discolor* ]	570	95%	0.0	69.63%	XP_ 028368416.1
AZIN2-like [ *Odocoileus virginianus texanus* ]	567	94%	0.0	67.15%	XP_ 020738443.1
ODC-like [ *Tupaia chinensis* ]	565	92%	0.0	71.32%	XP_ 006158213.2
Predicted low quality protein: AZIN2 [ *Ailuropoda melanoleuca* ]	564	99%	0.0	68.88%	XP_ 019660157.1
Tubulointerstitial nephritis antigen-like protein [ *Tupaia chinensis* ]	561	92%	0.0	71.32%	ELW48642.1
Predicted: AZIN2-like [ *Bison bison bison* ]	558	95%	0.0	67.73%	XP_ 010835381.1
Predicted low quality protein: AZIN2-like [ *Cebus* *capucinus imitator* ]	556	94%	0.0	68.14%	XP_ 017383214.1
Predicted low quality protein: ODC 2-like [ *Saimiri* *boliviensis boliviensis* ]	601	94%	0.0	67.83%	XP_ 010346508.1
Predicted: ODC2-like [ *Elephantulus edwardii* ]	553	93%	0.0	67.34%	XP_ 006895053.1
Low quality protein: AZIN2-like [ *Bos indicus x Bos taurus* ]	551	95%	0.0	66.59%	XP_ 027410042.1
AZIN2-like [ *Desmodus rotundus* ]	550	88%	0.0	71.35%	XP_ 024409968.1
Predicted: AZIN2-like [ *Sorex araneus* ]	548	95%	0.0	66.83%	XP_ 004603784.1
Predicted: AZIN2 [ *Bos mutus* ]	546	95%	0.0	67.24%	XP_ 005906296.1
Low quality protein: AZIN2-like [ *Bubalus bubalis* ]	546	92%	0.0	69.39%	XP_ 025120248.1
Predicted: AZIN2-like [ *Bos indicus* ]	545	95%	0.0	66.42%	XP_ 019839901.1
TPA: ODC 1-like [ *Bos taurus* ]	542	93%	0.0	66.83%	DAA32368.1
TINAL protein [ *Crocuta crocuta* ]	542	85%	0.0	70.03%	KAF0880816.1
ODC-like isoform X5 [ *Mus musculus* ]	541	62%	0.0	99.62%	XP_ 006503262.1
Low quality protein: AZIN2-like [ *Echinops telfairi* ]	637	96%	0.0	64.65%	XP_ 012860718.1
Hypothetical protein [ *Bos mutus* ]	537	93%	0.0	66.92%	MXQ79416.1
Low quality protein: AZIN2-like [ *Sapajus apella* ]	534	94%	0.0	67.16%	XP_ 032103474.1
Low quality protein: AZIN2-like [ *Bubalus bubalis* ]	532	95%	0.0	66.91%	XP_ 025120239.1
ODC2 [ *Bos mutus* ]	524	90%	0.0	66.75%	ELR48721.1
Low quality protein: AZIN2-like [ *Lagenorhynchus obliquidens* ]	516	92%	2 x 10^-179^	64.19%	XP_ 026966506.1
Low quality protein: AZIN2-like [ *Phoca vitulina* ]	509	92%	3 x 10^-177^	65.09%	XP_ 032270812.1
Low quality protein: AZIN2-like [ *Globicephala melas* ]	509	92%	8 x 10^-177^	63.43%	XP_ 030721326.1
AZIN2-like [ *Phascolarctos cinereus* ]	509	94%	1 x 10^-176^	61.69%	XP_ 020839122.1
Low quality protein: AZIN2-like [ *Neomonachus schauinslandi* ]	506	92%	8 x 10^-176^	64.59%	XP_ 021540090.1
Predicted low quality protein: AZIN2-like [ *Myotis* *brandtii* ]	503	92%	6 x 10^-174^	67.17%	XP_ 014396607.1
AZIN2 [ *Sarcophilus harrisii* ]	498	94%	3 x 10^-171^	59.20%	XP_ 031817379.1
AZIN2-like [ *Vombatus ursinus* ]	495	94%	8 x 10^-171^	60.20%	XP_ 027720538.1
AZIN2-like [ *Sarcophilus harrisii* ]	494	89%	2 x 10^-170^	60.63%	XP_ 031813151.1
Low quality protein: AZIN2-like [ *Leptonychotes weddellii* ]	489	93%	4 x 10^-169^	63.07%	XP_ 030884895.1
Low quality protein: AZIN2-like [ *Callorhinus ursinus* ]	488	91%	6 x 10^-169^	63.01%	XP_ 025712140.1

**Table 2:** 

Description	Total score	Query coverage	E value	Percent identity	Accession
ODC [ *Homo sapiens* ]	388	93%	2 x 10^-132^	48.52%	4ZGY_ A
ODC [ *Homo sapiens* ]	387	93%	1 x 10^-131^	48.52%	2ON3_ A
ODC [ *Homo sapiens* ]	387	93%	1 x 10^-131^	48.52%	2OO0_ A
ODC [ *Homo sapiens* ]	384	93%	7 x 10^-131^	48.28%	1D7K_ A
ODC [ *Mus musculus* ]	382	93%	3 x 10^-130^	46.32%	7ODC_ A
ODC [ *Trypanosoma brucei gambiense* ]	350	90%	1 x 10^-117^	45.57%	1NJJ_ A
ODC [ *Trypanosoma brucei* ]	350	90%	1 x 10^-117^	45.57%	1F3T_ A
ODC [ *Trypanosoma brucei brucei* ]	348	90%	1 x 10^-116^	45.32%	1SZR_ A
ODC [ *Trypanosoma brucei* ]	348	90%	1 x 10^-116^	45.32%	2TOD_ A
AZIN [ *Mus musculus* ]	323	91%	7 x 10^-107^	42.86%	3BTN_ A
AZIN 1 [ *Homo sapiens* ]	319	91%	2 x 10^-105^	42.86%	4ZGZ_ A
AZIN [ *Paramecium bursaria* Chlorella virus 1]	249	80%	1 x 10^-78^	38.24%	2NVA_ A
AZIN [ *Paramecium bursaria* Chlorella virus 1]	247	80%	1 x 10^-78^	37.94%	2NV9_ A
Lysine/Ornithine Decarboxylase [ *Selenomonas ruminantium* ]	205	83%	2 x 10^-61^	35.08%	5GJM_ A
Lysine/Ornithine Decarboxylase [ *Selenomonas ruminantium* ]	204	83%	5 x 10^-61^	35.08%	5GJO_ A

The structures with the highest identity were determined as Homo sapiens ornithine decarboxylase (PDB Code: 4ZGY) and Mus musculus ornithine decarboxylase (PDB Code: 7ODC) (Table 2). The homology between the query and the best hits were found to be 48.52% and 46.32%, respectively with a coverage of 93% for both. According to The National Centre for Biotechnology Information’s (NCBI) Conserved Domain Database (CDD) search, LDC belongs to Ornithine Decarboxylase subfamily (cd00622) of Type III Pyridoxal 5-phosphate (PLP) Dependent Enzymes family (cd06810). Members of this subfamily are known to contain PLP-binding triose phosphate isomerase (TIM) barrel domain and a C-terminal β -sandwich domain. They act as homodimers with active sites that lie at the interface between the TIM barrel domain of one subunit and the β -sandwich domain of the other subunit. Homodimer formation and the presence of PLP as a cofactor are required for catalytic activity [26].

### 3.2. Overall 3D structure of LDC contains 2 distinct domains

**Figure 3:**
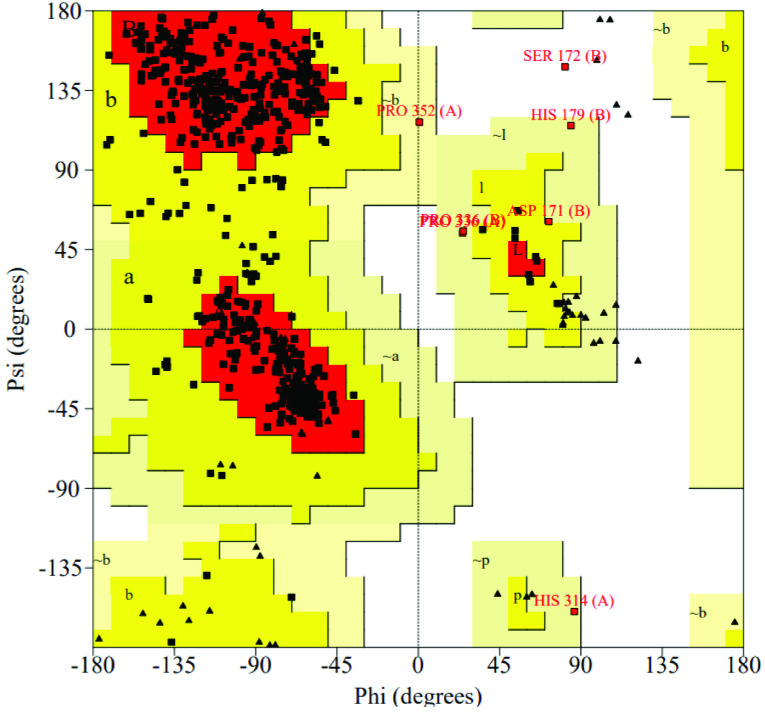
Ramachandran plot of LDC homology model.

The homology models of LDC enzyme were generated using SWISS-MODEL comparative modelling server (GMQE: 0.77; QMEAN: –0.56). The validation of the full model was performed with SAVES server. According to VERIFY results, the 81.61% of the residues have averaged 3D-1D score ≥ 0.2 for LDC. The overall quality factor was calculated with ERRAT and found to be 94.0%. The Ramachandran map was created by PROCHECK (Figure 3). The plot shows 90.4% amino acids (611) in the most favoured regions, 9.0% amino acids (61) in the additional allowed regions, 0.4% amino acids (3) in generously allowed regions, and only 1 amino acid (0.1%) in the disallowed regions, which suggested that the overall structure was reasonably good.

**Figure 4:**
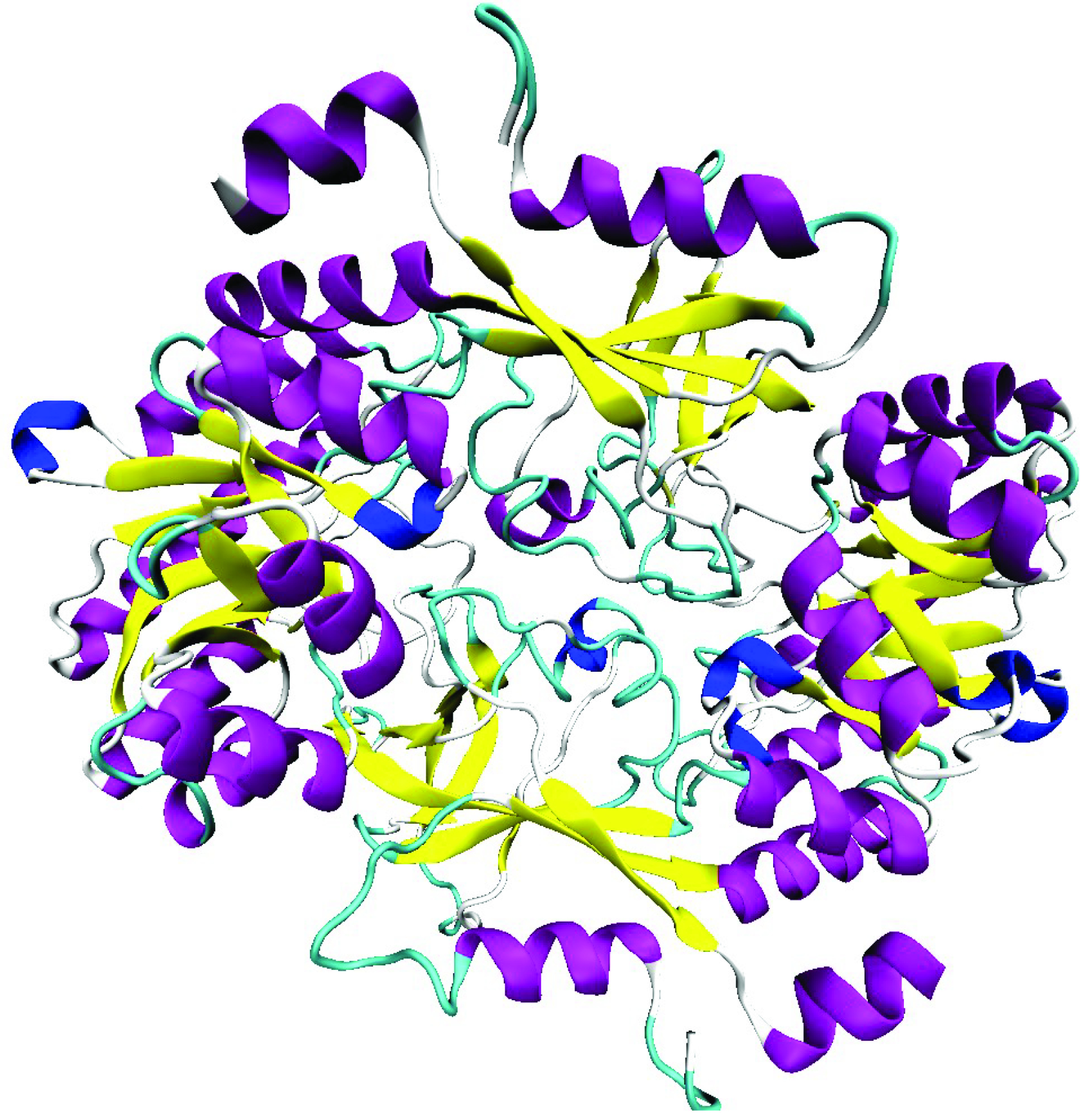
Overall 3D structure of LDC homodimer.

**Figure 5:**
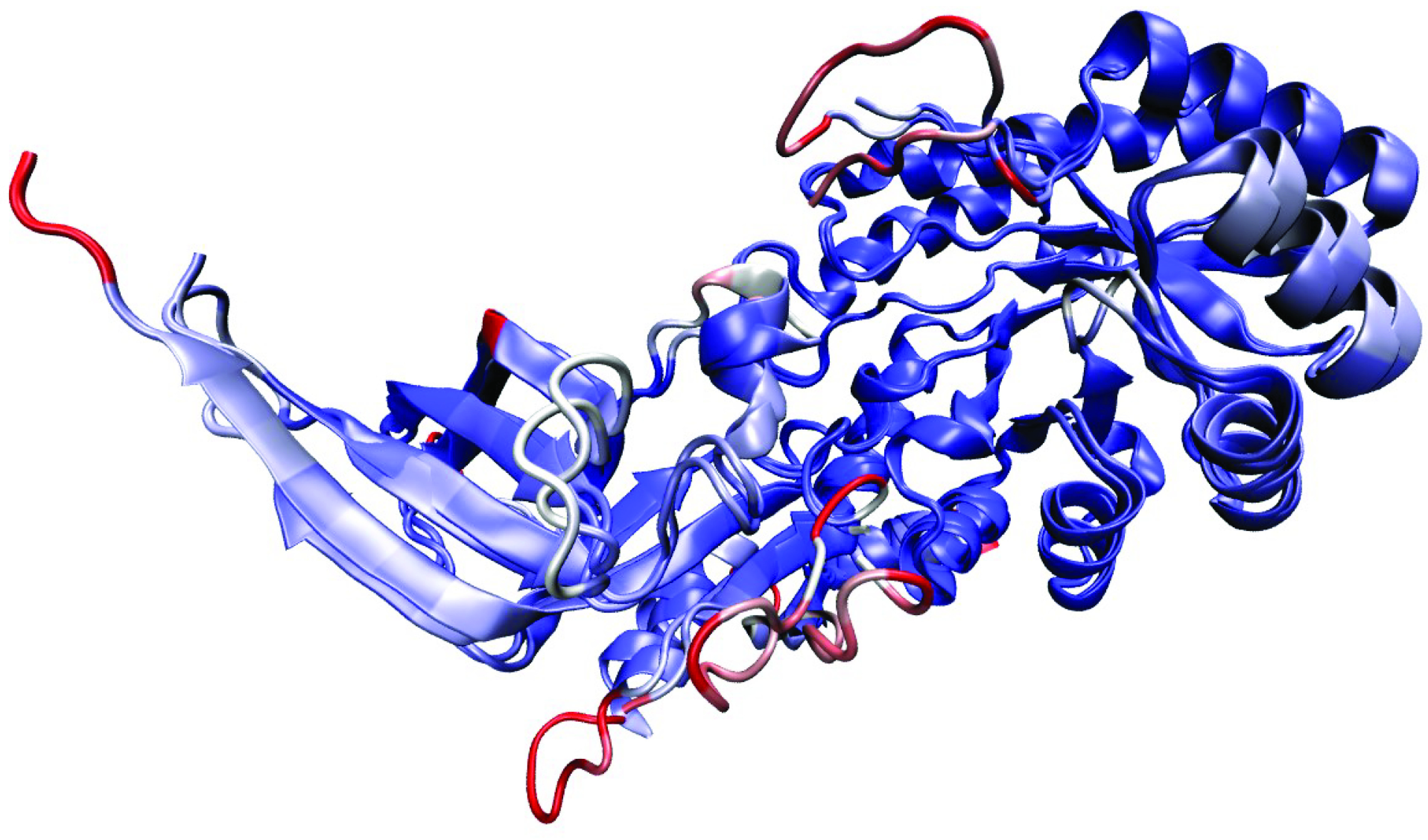
Structural alignment of Mus musculus LDC and Homo sapiens ODC subunits. Blue colour shows higher structural similarity whereas red shows lower.

The overall 3D structure of LDC homodimer is given in Figure 4. The structural similarity score between the model and the template was calculated with DALI Structure Comparison Server (Dali Z-Score = 51.4, RMSD = 1.6, %ID = 50) and visualized with MultiSeq (Figure 5) plugin of VMD. Similar to other PLP dependent enzymes of the same family, LDC contains 2 distinct domains. The N -terminal domain (26-287) adopts the (β /α)8 TIM barrel conformation whereas C-terminal domain (292-415) adopts a β -sandwich conformation. Similar to other members of Type III PLP-Dependent ODC subfamily (PLPDE_III_ODC), the enzyme forms a homodimer where the N -terminal domain of the one polypeptide chain interacts with the C-terminal domain of the other [27]. The functional homodimer structure contains 2 PLP binding site and binds 2 substrates. [28].

### 3.3. Active site of the LDC contains all 24 of the catalytically important residues of ODC

**Figure 6:**
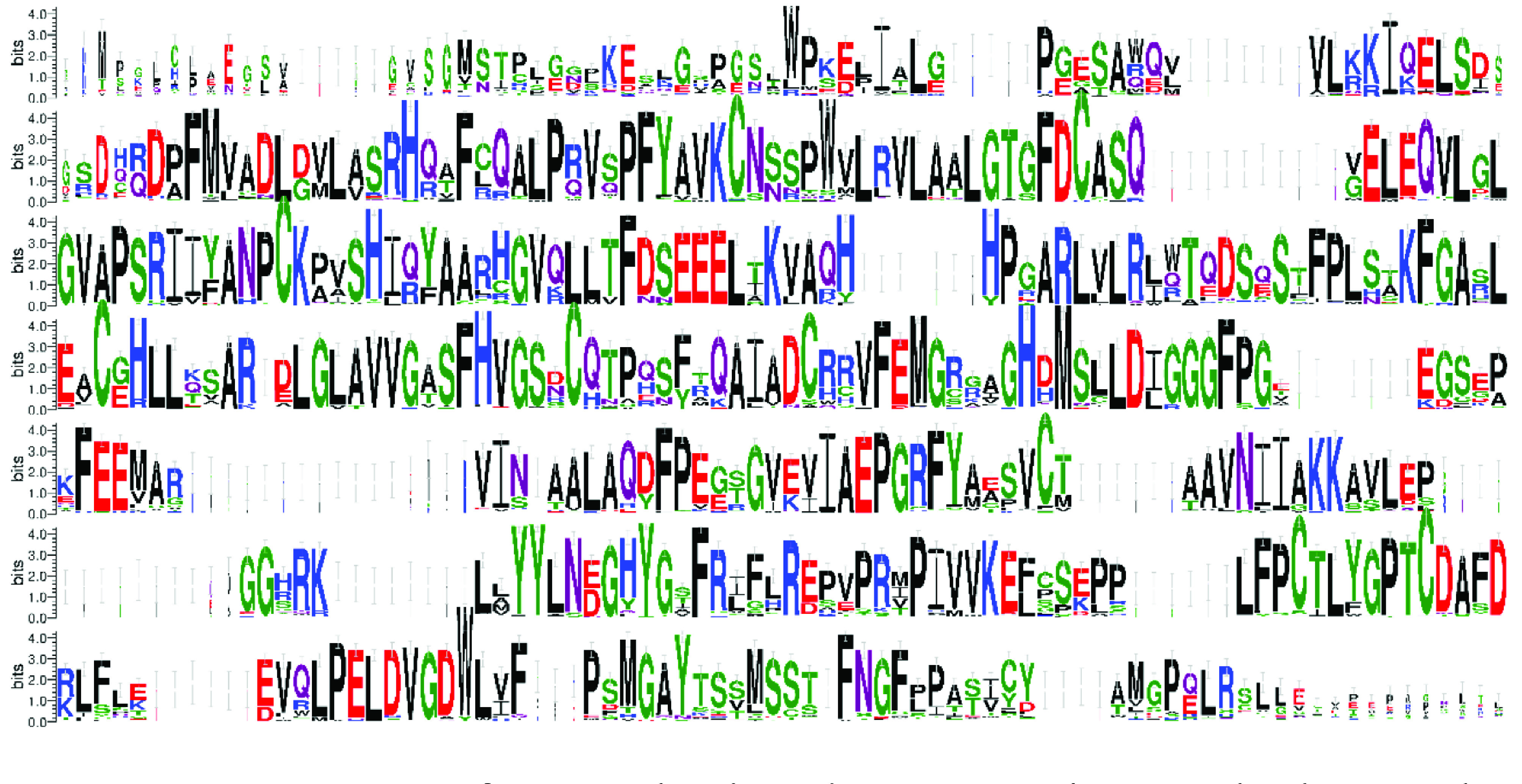
Sequence Logo of LDC against homolog structures in nonredundant protein structures.

LDC primary sequence is highly conserved (Figure 6). The active site of the enzyme is composed of residues from both subunits of the dimer. Conserved amino acid residues A54-V55-K56, D75, E81, E125-L126, R141, K156-F157-G158, H184-V185-G186-S187, G222-G223-G224, E261-P262-G263-R264, R309- D310, C338-D339-A340-Y341-D342, G366-A367-Y368, F402 are responsible for the active site formation and the covalent bonding of the PLP (Figure 6). K56 and C338 act as catalytic residues whereas the others interact with PLP and substrate. LDC enzyme contains all 24 of the catalytically important residues of ODC which suggests quite similar reaction mechanisms for both enzymes.

### 3.4. PLP interacts with the active site amino acids via H bonds and π interactions

PLP of the OCD family enzymes usually forms an internal aldimine bond with the N -terminal domain catalytic residue lysine, which is displaced by an external aldimine bond with the substrate during the reaction. The side chain of the second catalytic residue, C-terminal domain cysteine, rotates into the active site to control the protonation step that forms the product [28].

**Figure 7:**
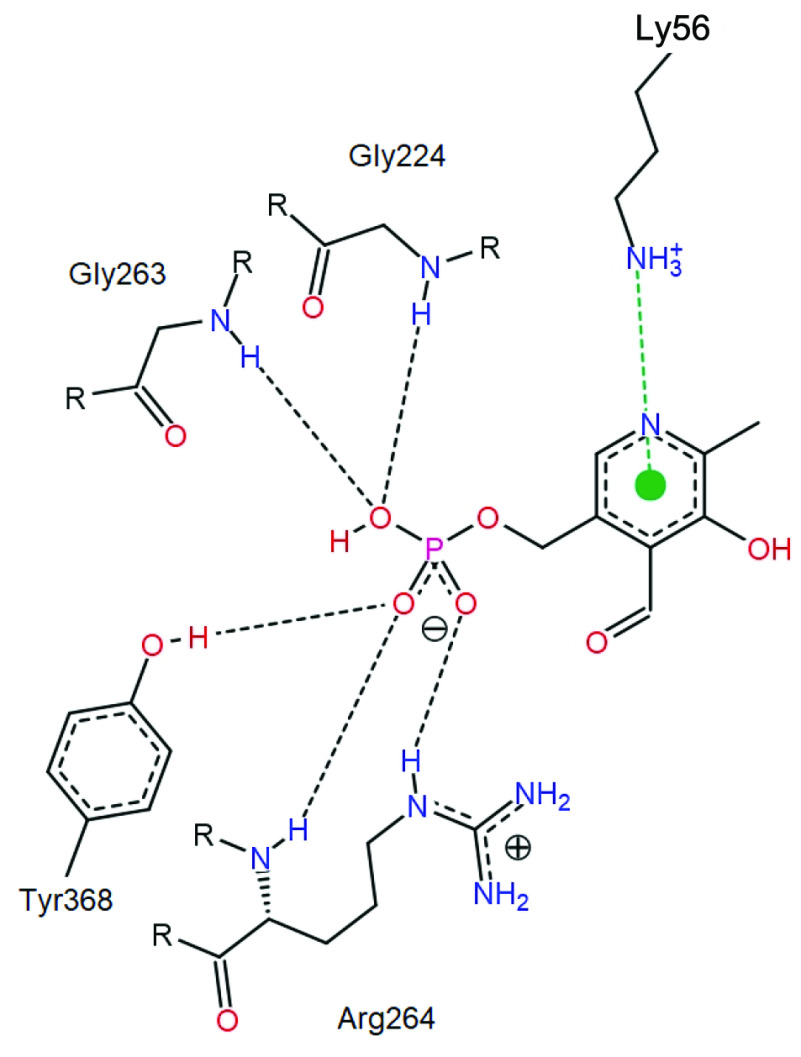
PoseView image of PLP in LDC active site based on the dock results. Black dashed lines indicate hydrogen bonds whereas green dashed lines indicate π-cation interactions.

Docking studies were performed by using NZ atom of ε-amino group of K56 as centre in a 10 Å^3^ grid box and allowing flexibility for side chains 3 Å of any atom of the ligand its reference binding mode. The cluster with the lowest energy within the dock results was used for evaluations. The PLP-enzyme interaction was visualized with PoseView (Figure 7). The results were similar to the interactions of the PLP with human and mouse ODCs (4ZGY, 2OO0, and 7ODC) which are good template structures according to BLAST results [29,30]. Imidazolium ring of H184 forms π-π stacking with the pyridine ring of the PLP and the ε-amino group of K56 was vertical to the pyridine plane forming a cation-π interaction with the pyridine ring and possibly an aldimine bond with the –C=O group of PLP depending on the reaction coordinate. These 2 amino acids effectively fixate the pyridine plane like an anvil and a hammer. The phosphate group of the PLP forms multiple H bonds with the side chain groups and the peptide bond nitrogen atoms of R264, G224, G263, S197, and Y368 amino acids of the (β /α)_8_ domain of the 1 subunit of the homodimer. Similar to K69 in human ODC (4ZGY), K56 amino group of LDC may form an H bond with oxygen of PLP–OH group depending on the reaction coordinate. The thiol group of C338 of the β -sandwich domain of the other subunit freely rotates moving into the active site in the presence of substrate.

### 3.5. LDC has a unique selectivity among other amino acid decarboxylases

**Figure 8:**
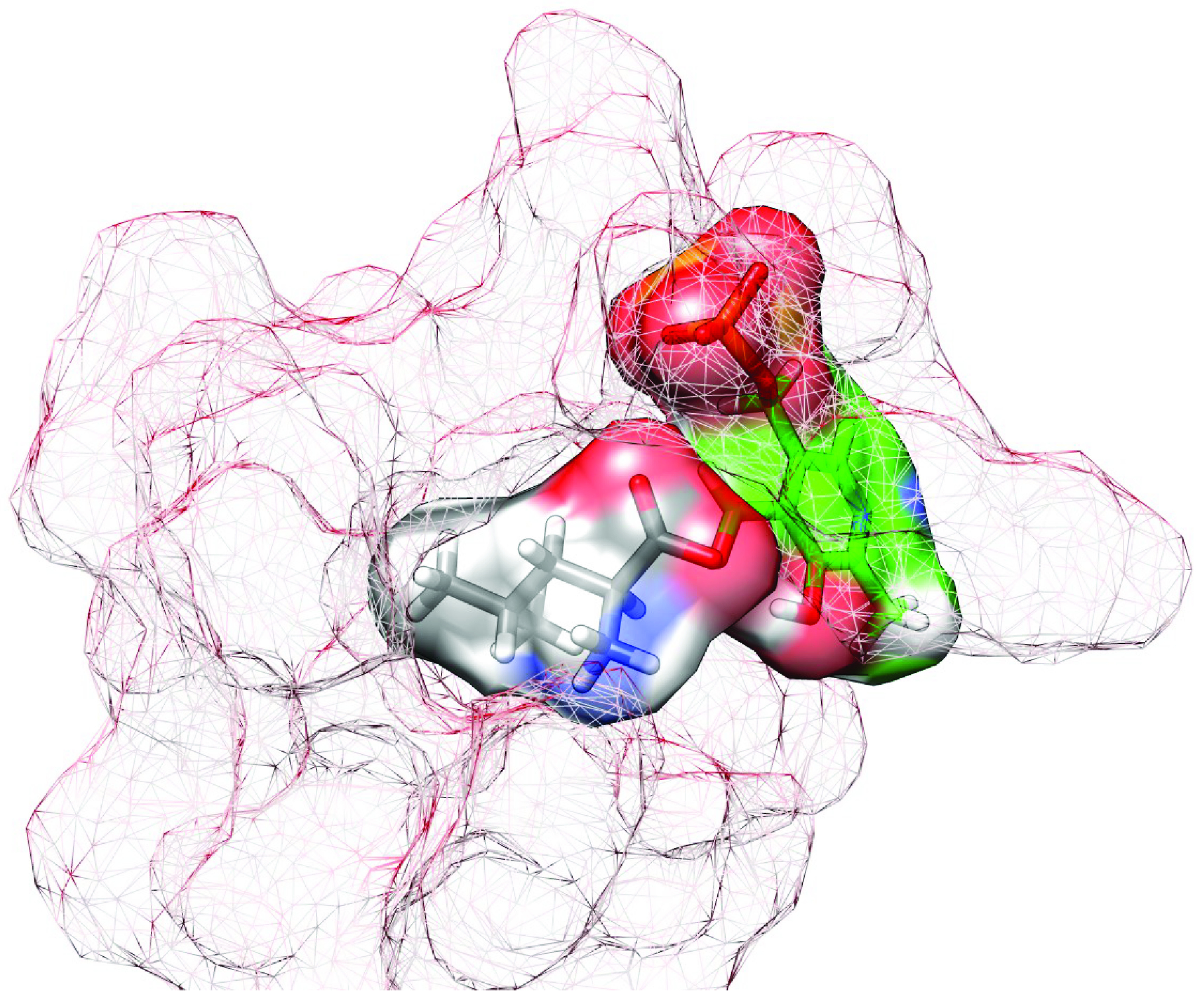
The active site of LDC with docked ligands PLP (green) and Leu (grey).

Leucine is an essential amino acid that is used in the biosynthesis of the proteins. Unlike valine and isoleucine, leucine is not branched at the β -carbon of the side chain. LDC is known to act on Leu but not on Val and Ile which lead the researchers to think the importance of the branching at β -carbon [3]. Leucine docking studies were performed by using NZ atom of ε-amino group of K56 as centred in a 10 Å^3^ grid box and allowing flexibility for side chains 3 Å of any atom of the ligand’s reference binding mode. The cluster with the secondlowest energy (highest full fitness) within the dock results were used for the evaluations instead of the lowest energy cluster to minimize the steric hindrances with PLP. According to dock results, the substrate forms H bonds with the side chains of H184, R264, and D339 amino acids. The side chains of D335 and the guanido group of R264 of the conserved –EPGR– motif sterically blocs the β -carbon of the amino acids which restrain β -branched amino acids from binding to the active site of the LDC. This motif is conserved within the ODC family enzymes which explains the similar β -carbon unbranched substrate selectivity of ODC and LDC. On the other hand, the active site of the LDC is relatively nonpolar and too small in length for positive charged and longer lysine and ornithine side chains which explain the perfect fit for Leu but no activity for lysine and ornithine (Figure 8).

## 4. Discussion

LDC is one of the 3 known ODC-related proteins together with AZIN1 and AZIN2 and suspected as a third AZIN until recently [2]. There are many LDC homologous sequences in databases that have high possibility to turn out to be other LDC isoenzymes.

LDC is a unique amino acid decarboxylase that acts only on leucine. Unlike similar enzymes, LDC acts on leucine but not ornithine, lysine, valine, or isoleucine and does not act as an AZIN. In this aspect, LDC is different from VDC, KDC, and ODC. The unique selectivity of the enzyme found to be directly related to its active site cleft which is relatively nonpolar and too small in length for positive charged and longer lysine and ornithine side chains. This active cleft sterically limits carbon chain length unlike ODC or KDC and β -carbon branching unlike VDC. The β -branched amino selectivity of the LDC found to be similar to ODC due to a conserved motif in active site.

Although the role and the metabolism of LDC and its product isopentylamine have not been fully understood yet, there are recent evidence on the possible effects of isopentylamine on gut microbiota [5] and type 2 diabetes mellitus [6].

As a biogenic primary amine, isopentylamine has been reported to be found in breastmilk and infant formulas, the amount of which in breastmilk, vary over time, increasing until the 1st week after delivery to a concentration similar to infant formulas. Isopentylamine has also been reported to be found in the meconium, and infant faeces however, by the 2nd week after birth, the amount of it were reported to be same in the faeces of those fed infant formula compared with those fed breast milk [5]. TAARs for isopentylamine have been reported to be found in the gut [31] and the diverse micro-organisms in adult faecal microbiota have been associated with the biogenic amines including isopentylamine [32]. These studies suggest that the ingested isopentylamine may contribute to facilitating healthy gut microbiota in infants and adults.

In a recent study, a branch of the TAAR family tree that is activated by isopentylamine, 2-phenylethylami ne, p-tyramine, and agmatine has been reported to increase intracellular cAMP significantly [6]. Thus, it has resulted in increased insulin secretion from INS-1 cells and primary mouse islets under normal conditions but not in glucolipotoxic conditions. According to authors, these findings may suggest that this subset of TAARs may be regulators of insulin secretion in pancreatic β -cells, as such they may be potential targets for treatment of type 2 diabetes. Isopentylamine is reported to be an endogenous agonist for TAAR3 [33] which belongs to this subset and therefore may have potential effects on type 2 diabetes mellitus metabolism.

Although the exact role of LDC in mammalian physiology is not known yet, the possible effects of its product, isopentylamine, makes this enzyme a worthy target for structural studies. The area of interest is completely novel and may provide many opportunities in the future. The role of LDC in mammalian physiology still waits to be discovered and although the crystal structure of the enzyme has not been determined yet, comparative modelling studies may still provide precious insights.
